# Different definitions of multimorbidity and their effect on prevalence rates: a retrospective study in German general practices

**DOI:** 10.1017/S146342362200010X

**Published:** 2022-04-06

**Authors:** Johannes Hauswaldt, Katharina Schmalstieg-Bahr, Wolfgang Himmel

**Affiliations:** 1 Department of General Practice, University Medical Centre, Göttingen, Germany; 2 Department of General Practice and Primary Care, University Medical Centre, Hamburg, Germany

**Keywords:** multimorbidity, practice visits, family practice, patient care management, electronic medical records

## Abstract

**Background:**

Multimorbidity is common among general practice patients and increases a general practitioner’s (GP’s) workload. But the extent of multimorbidity may depend on its definition and whether a time delimiter is included in the definition or not.

**Aims:**

The aims of the study were (1) to compare practice prevalence rates yielded by different models of multimorbidity, (2) to determine how a time delimiter influences the prevalence rates and (3) to assess the effects of multimorbidity on the number of direct and indirect patient contacts as an indicator of doctors’ workload.

**Methods:**

This retrospective observational study used electronic medical records from 142 German general practices, covering 13 years from 1994 to 2007. The four models of multimorbidity ranged from a simple definition, requiring only two diseases, to an advanced definition requiring at least three chronic conditions. We also included a time delimiter for the definition of multimorbidity. Descriptive statistics, such as means and correlation coefficients, were applied.

**Findings:**

The annual percentage of multimorbid primary care patients ranged between 84% (simple model) and 16% (advanced model) and between 74% and 13% if a time delimiter was included. Multimorbid patients had about twice as many contacts annually than the remainder. The number of contacts were different for each model, but the ratio remained similar. The number of contacts correlated moderately with patient age (r = 0.35). The correlation between age and multimorbidity increased from model to model up to 0.28 while the correlations between contacts and multimorbidity varied around 0.2 in all four models.

**Conclusion:**

Multimorbidity seems to be less prevalent in primary care practices than usually estimated if advanced definitions of multimorbidity and a temporal delimiter are applied. Although multimorbidity increases in any model a doctor’s workload, it is especially the older person with multiple chronic diseases who is a challenge for the GP.

## Introduction

Multimorbidity, defined as the presence of two or more conditions in one person (van den Akker *et al*., 1998; Le Reste *et al*., 2013; Kernick *et al*., 2017), places large demands on general practitioners’ (GPs’) workload and resource use, with increased rates of consultations and prescriptions (Hobbs *et al*., 2016; Cassell *et al*., 2018), even leading to GP burnout (Pedersen *et al*., 2020). To better understand the extent of these challenges, assessing the prevalence of multimorbidity in primary care is crucial. A systematic review on the prevalence of multimorbidity in primary care practices found low rates in younger patients but rates of 75% in patients aged 70 and older (Fortin *et al*., 2012). Recent studies found prevalence rates of even more than 80% in primary care populations (Cassell *et al*., 2018; Sinnige *et al*., 2015). However, in an editorial of this journal, Muna Adan and colleagues (Adan *et al*., 2020) criticised that prevalence studies are few, vary greatly in methodology and definition and, thus, affect the derived prevalence rates.

Naturally, the prevalence of multimorbidity depends on its definition, e.g., the number of chronic diseases included (Fortin *et al*., 2012; Prazeres and Santiago, 2018). But we know of only two studies that investigated the effect of different definitions of multimorbidity on prevalence rates for the same sample, one from Portugal with vague results (Prazeres and Santiago, 2018), one from Switzerland that only compared multimorbidity, defined as ≥2 and ≥3 chronic conditions (Excoffier *et al*., 2018). So, the effect of different definitions on prevalence rates is largely unknown.

Moreover, studies in the field of multimorbidity typically classify patients as being multimorbid or not for the remainder of their life. According to a Dutch study on prevalence patterns of chronic health problems (Vos *et al*., 2015), which uses a life span perspective to better understand trajectories of multimorbidity, it may be preferable to assess the prevalence and the burden of multimorbidity during defined time periods. Following this approach, a time delimiter for the definition of multimorbidity would be useful. In the primary care setting, this would mean that a physician should consider a patient, previously defined as being multimorbid, as a multimorbid patient only if multiple diseases are addressed or influence the current consultation.

Based on electronic medical records (EMRs) from German general practices, we compared practice prevalence rates yielded by four different models of multimorbidity for the same target population and studied how a time delimiter additionally influenced the results. The association of these different models with a patient’s annual number of direct and indirect patient contacts should reveal how multimorbidity affects a GP’s workload.

## Methods

### Context

All participating general practices are part of the German Statutory Health Insurance (SHI) system. For the definition of chronic diseases—a key aspect of most models of multimorbidity—we followed the billing rules of the SHI, based on a 3-month accounting period (annual quarter) for ambulatory care physicians. A patient’s diagnosis is considered a ‘chronic condition’, if the same diagnosis was also recorded in at least one of the three directly preceding quarters. This is called the M2Q-criterion, indicating reimbursement of at least one contact to the practice due to this condition in at least two quarters of the last 12 months (Busse *et al*., 2017).

### Study design

This retrospective observational study used EMR data from the MedViP study (Himmel *et al.*, 2006) and its spin-off projects for which German general practices provided information covering almost 13 years (55 quarters from the first quarter of 1994 up to the third quarter of 2007). Due to changes in privacy protection regulations (Hauswaldt *et al*. 2018), data from 2008 onwards could not be included in our analysis. For data preparation, analysis and reporting of the results, we used the RECORD checklist for REporting of studies Conducted using Observational Routinely-collected Data (Moher *et al*., 2014), see Appendix A.

### Participants

All GPs from our university department mailing list who answered an invitation letter were included in the MedViP study. For more information about this convenience sample and the database, see (Hauswaldt *et al*., 2013).

### Data extraction from EMRs

A patient from a participating practice was included in this analysis if he or she had a minimum of one direct or indirect patient contact, e.g. contact to the practice via phone and one diagnosis in at least two of the 55 annual quarters of the study period. This way, all included patients could theoretically develop or have a chronic disease by meeting the mentioned M2Q-criterion. A contact was defined as every calendar date (day) with an entry in a patient’s EMR, also including indirect communication with the GP or the practice staff (e.g. via phone) as well as back-office work for processing medical reports or laboratory data. We used this wide definition of contacts as a proxy for a doctor’s practice workload which is not alone influenced by personal patient contacts but also by administrative work to follow up patients.

Diagnoses were labeled according to the International Classification of Diseases (ICD-10) and truncated to their first three characters, that is, at ICD group level.

For each calendar year with a contact to the practice, a patient’s gender, age, number of contacts and all diagnoses in the respective year were extracted from the patient’s EMR. This data set of patient and calendar year (patient*year) was considered as ‘one case’ and is the unit of analysis.

Following different standard definitions in the literature, we compared four models of multimorbidity (Table 1) regarding their effect on the annual prevalence of multimorbidity and on the contact rates of multimorbid and non-multimorbid patients. The models differ in number and nature of included conditions, ranging from a simple definition, requiring only two diseases to an advanced definition requiring at least three chronic conditions.

**Table 1.** Models of multimorbidity and their definitions

Model	Label	Definition
Model 1	Multimorbidity by counting	At least 2 ICD codes were used for a patient in the year under study
Model 2	Multimorbidity according to EGPRN	At least one ICD code of a chronic condition plus at least one additional ICD code used for a patient in the year under study (Le Reste *et al*. 2013)
Model 3	Multimorbidity according to NICE	At least two ICD codes of chronic conditions were used for a patient in the year under study (Kernick *et al*., 2017)
Model 4	Multimorbidity according to Multicare	At least three ICD codes of chronic conditions were used for a patient in the year under study (Schäfer *et al*. 2014)

Note: ‘Chronic condition’ is defined according to the risk adjustment scheme of 2009 specified by the German SHI Federal Joint Commission chronic patients’ directive (for conditions meeting the so-called M2Q-criterion; see text).

According to these four models, we checked in two different ways whether a patient was multimorbid:

- Without a time delimiter: a patient was classified as being multimorbid for the rest of the study period (max. 55 quarters) after he or she met the criteria of a model at least once (‘no way back’).
- With a time delimiter: a patient was classified as being multimorbid for a calendar year if he or she met the criteria at least once during this year.

### Statistical analyses

We calculated means, standard deviations (SD), medians, ranges, and interquartile ranges (IQRs) for age, number of contacts and number of diagnoses at annual level, as descriptive statistics. The correlation of a patient’s number of contacts during each calendar year with age and with the different models of multimorbidity was calculated by Pearson’s correlation coefficient r or the point biserial correlation coefficient (r_pb_), as appropriate.

For all statistics, we used Stata 16.1 of Stata Corp LLC, Texas, USA.

## Results

### Patient sample

**Figure 1.**
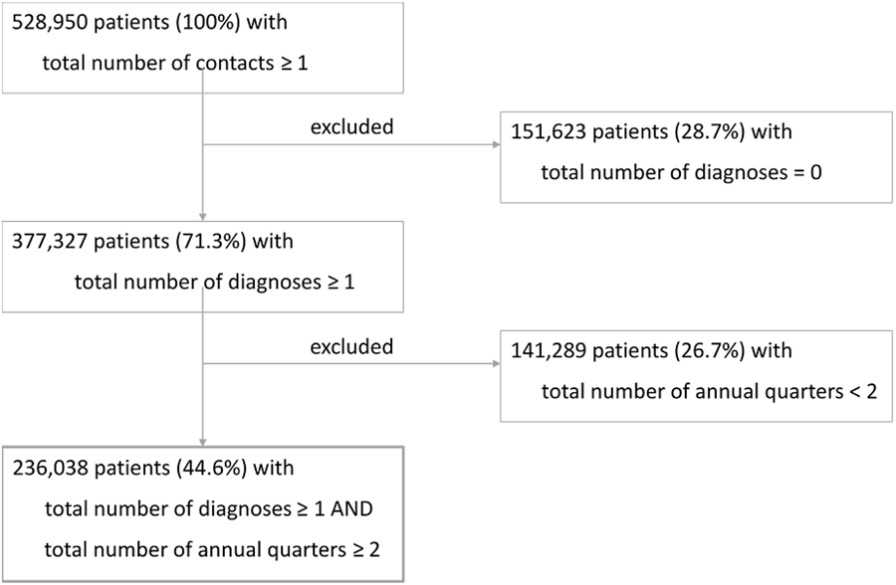
Flow chart of patient sample.

The raw data set comprised 528 950 patients. Of these, 292 912 patients (55.4%) were excluded because a diagnosis was missing (28.7%) or they were only seen in one annual quarter between 1994 and 2007 (26.7%). This resulted in a valid sample of 236 038 patients (44.6%) from 142 general practices (Figure 1). On average, study patients were 49 years old at the end of study (SD: 23.6, median: 47), and 54% (127,881/236,038) were female.

Since many of the 236 038 patients contributed data to several calendar years, the following analyses are—according to our case definition—based on 612 278 cases (patient*years).

### Prevalence of multimorbidity

Annual multimorbidity rates strongly depended on the definition of multimorbidity and to a lesser degree on the use of a time delimiter:

- Without a time delimiter, meaning to propagate a patient’s multimorbid status into the following years of observation until the end of study period, the proportion of multimorbid patients ranged from 84%, according to the simple definition of model 1, to 46%, 26% and 16%, according to models 2, 3 and 4, respectively (Figure 2).
  **Figure 2.**
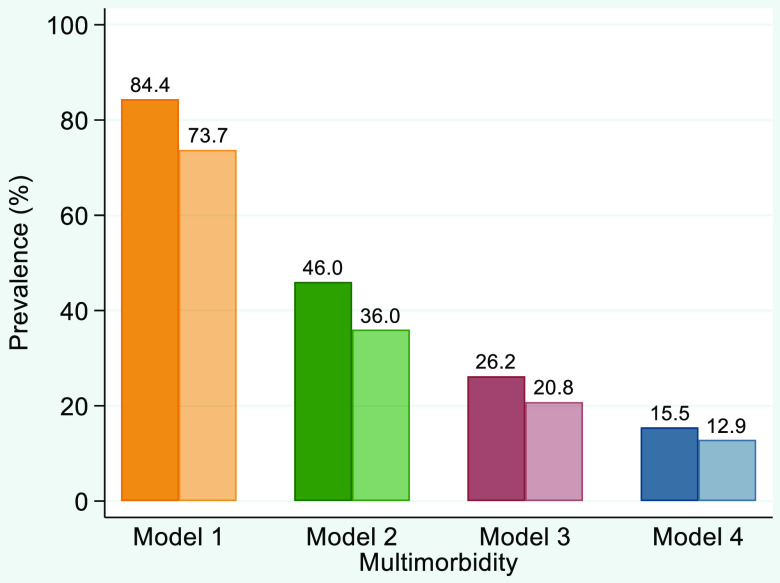
Average multimorbidity rates (percent) of four multimorbidity models, without (opaque colour) and with (transparent colour) a time delimiter, 612 278 cases (patient*years).

With a time delimiter, that is strictly studying patient’s data on annual base, nearly three-quarters (74%) of the cases were multimorbid, according to model 1, and 13%, according to model 4 (Figure 2).

Female patients were always more likely to be multimorbid than male patients, but the gender ratio remained stable across all four models of multimorbidity, with and without time delimiter. Looking at the data of any calendar year, multimorbid patients were older than non-multimorbid patients, with the difference increasing from model 1 to model 4 (Table 2).

**Table 2.** Number of cases, patients’ gender, age and average annual number of practice contacts, in four models of multimorbidity, without and with a time delimiter, 612 278 cases (patient*years)

		Model 1			Model 2			Model 3			Model 4		
		multimorbid			multimorbid			multimorbid			multimorbid		
		no	yes	corr*	no	yes	corr*	no	yes	corr*	no	yes	corr*
without time delimiter	Cases (N)	95,759	516,519		330,716	281,562		451,978	160,300		517,608	94,670	
	Female (%)	52.2	56.6	0.04	53.5	58.8	0.06	54.4	60.4	0.05	54.9	61.4	0.05
	Average age (y)	39.0	43.9	0.08	39.9	46.9	0.15	40.0	51.9	0.22	40.5	57.3	0.28
	Average annual number of contacts (N)	4.2	9.2	0.17	6.2	10.9	0.22	7.1	11.9	0.20	7.6	12.8	0.18
with time delimiter	Cases (N)	161,019	451,259		391,949	220,329		485,117	127,161		533,502	78,776	
	Female (%)	52.2	57.3	0.05	53.9	59.7	0.06	54.7	60.7	0.05	55.1	61.6	0.05
	Average age (y)	39.0	44.5	0.10	39.9	48.8	0.18	40.0	54.9	0.26	40.6	60.3	0.28
	Average annual number of contacts (N)	4.4	9.8	0.22	6.4	11.8	0.25	7.3	12.6	0.20	7.7	13.3	0.18

Note: corr* = point biserial correlation coefficient (r_pb_).

### Multimorbidity and contacts to the practice

On average, patients had 8.4 contacts to the practice per year (SD: 10.7; median: 5; IQR: 2-10). Female patients had more contacts per year (8.9; SD: 11,1) than men (7.7; SD: 10.1). However, gender affected contacts far less than multimorbidity. Multimorbid patients had about twice as many contacts annually than those not categorised as multimorbid, e.g. 9.8 vs. 4.4 in model 1 or 13.3 vs. 7.73 in model 4 (Table 2). The number of contacts were different for each model, but the ratio of multimorbid vs. non-multimorbid patients remained similar.

The correlations between age, patient’s contacts to the practice and multimorbidity showed the following patterns. The overall patients’ annual number of contacts correlated moderately with their age (r = 0.35). The correlation between age and multimorbidity was rather low for model 1 (around 0.1) and increased from model to model up to 0.28 in model 4 while the correlations between the number of contacts and multimorbidity varied around 0.2 and remained nearly the same in all four models (Table 2) regardless whether a time delimiter was included or not.

## Discussion

### Summary of main findings

Depending on the four different models of multimorbidity and whether or not a time delimiter was included, the annual practice prevalence of multimorbidity ranged between 13% and 84%. Multimorbid patients had about twice as many contacts than those not being categorised as being multimorbid, irrespective of the underlying model of multimorbidity.

### Strengths and limitations

This study is one of the few studies to compare different multimorbidity models regarding their effect on prevalence rates and a patient’s annual number of contacts. A further strength of our study is the decision to introduce a ‘temporal delimiter’ for chronic diseases and thus to allow for changes in a patient’s status, while other studies on multimorbidity typically classify patients globally as being either multimorbid or not. From a GP’s viewpoint, this is especially important when a simple model of multimorbidity is applied (model 1 including at least 2 ICD-codes) as diagnoses such as upper respiratory infection and back pain might be short-lived and do not warrant to permanently label a patient as multimorbid.

When categorising a patient’s diagnosis in an annual quarter as a ‘chronic condition’ according to the M2Q criterion, we did not compensate for left-censoring at the beginning of a patient’s total observation period. This may lead us to err on the conservative side for his or her initial three annual quarters.

A limitation of our study was the use of old data. However, the aim was not to present new trends in multimorbidity but to determine its impact on the use of health care resources and possible effects on a GP’s workload.

### Comparison with literature

Multimorbidity is considered to significantly impact general practice, reflected by a large number of studies (van den Akker *et al*., 1998; Fortin *et al*., 2005; Fortin *et al*., 2012; Roso-Llorach *et al*., 2018; Ricci-Cabello *et al*., 2015) Compared to reported prevalence rates of more than 50% (Prazeres and Santiago, 2018) or even more than 80% or 90%, at least for older patients (Sinnige *et al*., 2015; Roso-Llorach *et al*., 2018), our results indicated that the extent of multimorbidity may be overestimated in many studies. Only when using a wide definition, which just requires the co-incidence of any two conditions for a patient to be categorised as multimorbid (model 1), nearly three-quarters of all patients per year met the criteria in our sample. By applying advanced definitions of multimorbidity (models 2, 3 or 4), no more than 36%, 21% or just 13% of patients per year fell into this category. An Australian study on musculoskeletal conditions among working-age adults also concluded that, depending on definition and threshold, multimorbidity is either rare or endemic (Lowe *et al*., 2017).

It may sound trivial that the prevalence of multimorbidity is a matter of definition but the effect of the four models on practice prevalence was striking and unexpected in this magnitude. Using two rather similar definitions of multimorbidity (≥2 vs. ≥3 chronic conditions), the Swiss study (Excoffier *et al*., 2018) found an in-between range of about 20 percentage points, compared to the large range of about 70 percentage points in our study with four very distinguished models, also including acute diseases (model 1 and 2). Moreover, the time delimiter that allowed us to re-label patients from being multimorbid to non-multimorbid also played a role, albeit a limited one. Our decision to include a time delimiter, to date unique in the literature, may be justified since GPs may perceive multimorbidity only if multiple diseases are addressed or influence the current consultation.

We considered a patient’s number of contacts per year as a proxy for a GP’s workload, similar to a study on consultation rates in general practice in England (Mukhtar *et al*., 2018) and a study on daily stress and fatigue (Warner, 2017). According to a Dutch study on multimorbidity and health care utilisation, patients with multiple chronic diseases have about 18 contacts per year, those with one chronic disease 12 contacts and those without any chronic disease 6 contacts (van Oostrom *et al*., 2014). We found that multimorbid patients visited their GP about twice as much as the remainder, but on average no more than 13 times per year—even when patients met the criteria of an advanced definition (model 4) and a time delimiter was applied.

The increase of a GP’s workload by multimorbidity, as expressed in a doubling of contacts, is further complicated by an increase of contacts by patient age. Multimorbidity has been consistently associated with age (Violan *et al*., 2014), and studies in primary care confirm that older patients consulted their GP much more than younger patients (Mukhtar *et al*., 2018; Harrison *et al*., 2019). Our study adds to these findings insofar that the association between contacts and multimorbidity seems to be nearly the same throughout all four models, but the effect of age increases from the simpler models to the more advanced. Or, to put it in other words, a GP’s workload increases for patients with more than one disease, but it is especially the older patient with three or more chronic diseases who may consult the GP 13 times a year.

### Implications for practice and future research

We do not want to downplay multimorbidity as a challenge and a useful concept in primary care, but it often seems to be overestimated. Even our advanced model of multimorbidity may overestimate the prevalence of multimorbidity in general practice. More comprehensive definitions of multimorbidity and/or complex problems may mirror a GP’s workload to a greater extent than a patient’s number of diagnoses. These definitions may include, for example, a ‘bio-psychosocial factor’, as suggested by a European General Practice Research Network (EGPRN) working group (Le Reste *et al*., 2013) that generated a research agenda on ‘Multimorbidity in Family Practice’ (Le Reste *et al*., 2015). Future research should also help GPs how to balance potentially conflicting evidence concerning the single disease entities involved and the tightrope between partially contradictory guidelines for each disease (Boyd *et al*., 2005; Boyd and Fortin, 2010).

## Conclusion

Multimorbidity, if properly defined and including a temporal delimiter as well, applies to a smaller percentage of annual patient–physician encounters in primary care than usually reported. Although multimorbidity increases, in any case, a doctor’s workload, it is especially the older person with multiple chronic diseases who is a challenge for the GP.

## Supplementary material

For supplementary material accompanying this paper visit http://doi.org/10.1017/S146342362200010X. click here to view supplementary material
