# Supplementary material for: Different definitions of multimorbidity and their effect on prevalence rates: a retrospective study in German general practices
Source: Prim Health Care Res Dev. 2022 Apr 6;23:e25. doi: 10.1017/S146342362200010X (PMC8991077; doi:10.1017/S146342362200010X)
Supplement: Supplementary file 1 [file S146342362200010Xsup001.docx]

Benchimol, Eric I.; Smeeth, Liam; Guttmann, Astrid; Harron, Katie; Moher, David; Petersen, Irene et al. (2015): The REporting of studies Conducted using Observational Routinely-collected health Data (RECORD) statement. In: PLoS medicine 12 (10), e1001885. DOI: 10.1371/journal.pmed.1001885.

**Title and Abstract**

1 (a) Indicate the study’s design with a commonly used term in the title or the abstract

Different definitions of multimorbidity and their effect on prevalence rates

A retrospective study in German general practices

(b) Provide in the abstract an informative and balanced summary of what was done and what was found

RECORD ITEM 1.1: The type of data used should be named in the title or abstract. Where possible, the names of the databases used should be included.

- Administrative and medical data routinely collected day-to-day in general practices, electronic medical records (EMR)

RECORD ITEM 1.2: If applicable, the geographic region and time frame within which the study took place should be reported in the title or abstract.

- Germany, 1994 to 2007

RECORD ITEM 1.3: If linkage between databases was conducted for the study, this should be clearly stated in the title or abstract.

- (No linkage of databases)

**Introduction**

Background rationale

2 Explain the scientific background and rationale for the investigation being reported

- Several definitions for multimorbidity exist. It remains unclear how these definitions differ in their prevalence and what multimorbidity of a patient means in terms of patient’s burden of disease or workload to practice and physician

Objectives

3 State specific objectives, including any prespecified hypotheses

- To assess the annual percentage of multimorbid patients in primary care
- To assess the effects of multimorbidity on the patients’ number of practice contacts as an indicator of doctors’ workload.

**Methods**

Study Design

4 Present key elements of study design early in the paper

- Purely quantitative approach
- Basic measure for all variables is a patient’s year, from 1994 until 2007

Setting

5 Describe the setting, locations, and relevant dates, including periods of recruitment, exposure, follow-up, and data collection

- Germany, 1994 - 2007, general practices, electronic medical records.
- Re-utilization of research data from previous study

Participants

6 (a) Cohort study - Give the eligibility criteria, and the sources and methods of selection of participants. Describe methods of follow-up

Case-control study - Give the eligibility criteria, and the sources and methods of case ascertainment and control selection. Give the rationale for the choice of cases and controls

Cross-sectional study - Give the eligibility criteria, and the sources and methods of selection of participants

- All patients of general practice which had at least one contact and one diagnosis in at least two annual quarters.
- Convenience sample of practices.

(b) Cohort study - For matched studies, give matching criteria and number of exposed and unexposed

Case-control study - For matched studies, give matching criteria and the number of controls per case

- (not applicable)

RECORD 6.1: The methods of study population selection (such as codes or algorithms used to identify subjects) should be listed in detail. If this is not possible, an explanation should be provided.

- All patients of a practice were included which had at least one contact and one diagnosis in at least two annual quarters.

RECORD 6.2: Any validation studies of the codes or algorithms used to select the population should be referenced. If validation was conducted for this study and not published elsewhere, detailed methods and results should be provided.

- Secondary analysis of routine data from EMR has been performed successfully for several times, yielding sufficient external validation.

RECORD 6.3: If the study involved linkage of databases, consider use of a flow diagram or other graphical display to demonstrate the data linkage process, including the number of individuals with linked data at each stage.

- (not applicable)

Variables

7 Clearly define all outcomes, exposures, predictors, potential confounders, and effect modifiers. Give diagnostic criteria, if applicable.

- Number of contacts per patient and year
- Number of diagnoses per patient and year
- Number of chronic diseases (chronic conditions) per patient and year
- Patient’s gender and age

RECORD 7.1: A complete list of codes and algorithms used to classify exposures, outcomes, confounders, and effect modifiers should be provided. If these cannot be reported, an explanation should be provided.

- For data analysis we used STATA 16.1

Data sources/measurement

8 For each variable of interest, give sources of data and details of methods of assessment (measurement). Describe comparability of assessment methods if there is more than one group.

- Any patient contact to the practice observed from electronic practice management system was taken.
- Any ICD code was truncated to leading 3 characters (ICD group level)
- Number of contacts and number of diagnoses were aggregated per patient at annual level
- If at least one contact and one diagnosis was found in at least two annual quarters, patient was included into this study

Bias

9 Describe any efforts to address potential sources of bias

- Selection bias due to convenience sampling of practices can not be excluded.
- All patients of a practice who met inclusion criteria („whole case analysis“) were analysed
- Retrospective analysis of EMR without intervention

Study size

10 Explain how the study size was arrived at

- Original raw data consisted of 18 mio datasets, from 528,950 patients, from 166 practices
- Inclusion of contact and diagnosis data left 3.3 mio data sets for this analysis
- Aggregation at patient quarter level resulted in 1.3 mio datasets
- Exclusion of datasets with missings for gender or age resulted in 612,278 whole datasets (cases, patient*year), with 236,038 patients from 144 practices; the study dataset

Quantitative variables

11 Explain how quantitative variables were handled in the analyses. If applicable, describe which groupings were chosen, and why.

- Patient’s gender: male=0 | female=1
- Patient’s age: days between birthdate and first day of annual quarter
- Number of contacts: natural numbers (positive integers)
- Number of diagnoses: natural numbers (positive integers)
- Number of chronic conditions: natural numbers (positive integers and zero)

Statistical methods

12 (a) Describe all statistical methods, including those used to control for confounding

- Descriptive statistics: means, standard deviations (SD), medians, ranges, and interquartile ranges (IQRs) for age, number of contacts, and number of diagnoses at annual level
- Analytical statistics: correlation of a patient’s number of contacts during each calendar year with age was calculated by Pearson’s correlation coefficient r
- Analytical statistics: correlation of a patient’s number of contacts during each calendar year with the different models of multimorbidity was calculated by the point biserial correlation coefficient (rpb)

(b) Describe any methods used to examine subgroups and interactions

- For patients‘ gender and average age, results are shown being multimorbid or non-multimorbid

(c) Explain how missing data were addressed

- Datasets with missings were excluded („whole data analysis“)

(d) Cohort study - If applicable, explain how loss to follow-up was addressed

Case-control study - If applicable, explain how matching of cases and controls was addressed

Cross-sectional study - If applicable, describe analytical methods taking account of sampling strategy

- Re-utilization of research data from previous study, Germany, 1994 - 2007, general practices, electronic medical records.
- Convenience sample of practices.
- All patients of a practice were included which had at least one contact and one diagnosis in at least two quarters.

(e) Describe any sensitivity analyses

- (n.a.)

Data access and cleaning methods

RECORD 12.1: Authors should describe the extent to which the investigators had access to the database population used to create the study population.

- Raw data were anonymized with respect to practices and patients
- Authors had no access to the general practices or their patients.

RECORD 12.2: Authors should provide information on the data cleaning methods used in the study.

- Flow chart of data preparation:

(1) Original BDT data

gained between 2002 and 2008, MedViP project and its consecutive projects,

datasets from 166 German general practices, about 1 mio patients.

„BDT“ stands for „*Behandlungsdatentransfer*“, a mandatory software interface to be expected in all German electronic practice managment systems (PMS).

Original BDT data format, as defined in 1994, is one single text data collection from all EMR of a PMS consisting of alfanumerics, together with line feed (LF) and carriage return (CR) markers for separating information flow into data lines (datasets).

These data were erased after end of projects due to legal regulation in Germany.

(2) Raw data set

prepared from (1) original BDT data.

18,869,541 datasets, 8 variables, 155 practices, 472,829 patients.

These data are not to be disclosed to third parties

according to projects' ethical statements comprising privacy protection contracts with the initially submitting practices.

(3) Raw data set for this study

prepared from (2) raw data set in may 2019, after aggregation on annual quarter base, following inclusion criteria as described in main text.

1,334,556 datasets, 19 variables, 145 practices, 226,256 patients.

(4) Study data set for this study

prepared from (3) raw data set for this study, after exclusion of datasets incomplete for gender and/or age of patient.

612,278 datasets, 25 variables, 144 practices, 236,038 patients.

Linkage

RECORD 12.3: State whether the study included person-level, institutional-level, or other data linkage across two or more databases. The methods of linkage and methods of linkage quality evaluation should be provided.

- (No data linkage)

Results

Participants

RECORD 13.1: Describe in detail the selection of the persons included in the study (i.e., study population selection) including filtering based on data quality, data availability and linkage.The selection of included persons can be described in the text and/or by means of the study flow diagram.

- All patients of a practice were included which had at least one contact and one diagnosis in at least two quarters.

Descriptive data

14 (a) Give characteristics of study participants (e.g., demographic, clinical, social) and information on exposures and potential confounders

- (No additional information available)

(b) Indicate the number of participants with missing data for each variable of interest

- See above, RECORD 12.2

(c) Cohort study - summarise follow-up time (e.g., average and total amount)

- (Not applicable)

Outcome data

15 Cohort study - Report numbers of outcome events or summary measures over time

Case-control study - Report numbers in each exposure category, or summary measures of exposure

Cross-sectional study - Report numbers of outcome events or summary measures

- See Table 2 of main text

**Main results**

16 (a) Give unadjusted estimates and, if applicable, confounderadjusted estimates and their precision (e.g., 95% confidence interval). Make clear which confounders were adjusted for and why they were included

- See Table 2 main text

(b) Report category boundaries when continuous variables were categorized

- (Not applicable)

(c) If relevant, consider translating estimates of relative risk into absolute risk for a meaningful time period

- (Not applicable)

Other analyses

17 Report other analyses done— e.g., analyses of subgroups and interactions, and sensitivity analyses

- Density plots and boxplots for all independent variables (results not shown)

**Discussion**

Key results

18 Summarise key results with reference to study objectives

- Different multimorbidity definitions result in different percentages of all patient population, identified from electronic medical records of 144 German general practices, 1994 to 2007
- Number of patient’s contacts is influenced by multimorbidity only moderately. Patient’s age has greater impact.

Limitations

19 Discuss limitations of the study, taking into account sources of potential bias or imprecision. Discuss both direction and magnitude of any potential bias

- Practices‘ selection is a convenience sample and thus is not representative for German practices
- Primary data are from 1994 to 2007. Data from 2008 onwards were not included in this analysis, due to changes in data protection regulations.

RECORD 19.1: Discuss the implications of using data that were not created or collected to answer the specific research question(s). Include discussion of misclassification bias, unmeasured confounding, missing data, and changing eligibility over time, as they pertain to the study being reported.

- „Practice contact“ meant any patient contact as derived from EMR and included not only contact to the physician, but to any practice professional as well as indirect contact, e.g. integrating laboratory results or other information concerning the patient.
- „Diagnosis“ from ICD code may be just transferred over time acoording to procedural habit of the practice. ICD codes were truncated to leading 3 characters (ICD group level)

Interpretation

20 Give a cautious overall interpretation of results considering objectives, limitations, multiplicity of analyses, results from similar studies, and other relevant evidence

- To our knowing, this study is the first which operationalises four common definitions for multimorbidity on a temporal base, und thus makes them comparable.
- The basic unit for analysis was the patient’s calendar year. Following the German billing system for Statutory Health Insurance (SHI) practices, the basic unit for a patient’s diagnoses and his/her having a chronic condition was the annual quarter
- A patient’s diagnosis was categorised as being a ‘chronic condition’, if the same diagnosis in addition was observed in at least one of the three directly preceding quarters. This is in line with a common German definition used with the risk adjustment scheme (Risikostrukturausgleich), introduced in 2009 to balance health risks and costs between the SHI companies.

Generalisability

21 Discuss the generalisability (external validity) of the study results.

- Generalisability of this study is limited due convenience sampling of practices and time period, 1994 unti 2007
- The study defines methods and operationalising procedures which allow for comparable future studies

**Other information**

Funding

22 Give the source of funding and the role of the funders for the present study and, if applicable, for the original study on which the present article is based.

- In parts by Deutsche Forschungsgemeinschaft (DFG), Fördernummern HU 1587/2-1, HO 1937/7-1, RI 1000/7-1, YA 191/8-1, KR 1093/10-1 vom 29.04.2016

Accessibility of protocol, raw data, and programming code

RECORD 22.1: Authors should provide information on how to access any supplemental information such as the study protocol, raw data, or

programming code.

- Study protocol is available from JH
- Raw data are deleted according to legal regulation
- No specific programming code has been used outside of STATA 16.1 features

(20210506jh)
